# Application of Electrochemical Sensors Based on Carbon Nanomaterials for Detection of Flavonoids

**DOI:** 10.3390/nano10102020

**Published:** 2020-10-14

**Authors:** Jinchun Hu, Zhenguo Zhang

**Affiliations:** Shandong Provincial Key Laboratory of Animal Resistance Biology, Key Laboratory of Food Nutrition and Safety, College of Life Sciences, Shandong Normal University, Jinan 250014, China; hujinchun_369@163.com

**Keywords:** flavonoids, food and drug homologous substances, electrochemical sensors, carbon nanomaterials, detection

## Abstract

Flavonoids have a variety of physiological activities such as anti-free radicals, regulating hormone levels, antibacterial factors, and anti-cancer factors, which are widely present in edible and medicinal plants. Real-time detection of flavonoids is a key step in the quality control of diverse matrices closely related to social, economic, and health issues. Traditional detection methods are time-consuming and require expensive equipment and complicated working conditions. Therefore, electrochemical sensors with high sensitivity and fast detection speed have aroused extensive research interest. Carbon nanomaterials are preferred material in improving the performance of electrochemical sensing. In this paper, we review the progress of electrochemical sensors based on carbon nanomaterials including carbon nanotubes, graphene, carbon and graphene quantum dots, mesoporous carbon, and carbon black for detecting flavonoids in food and drug homologous substances in the last four years. In addition, we look forward to the prospects and challenges of this research field.

## 1. Introduction

With the development of the economy and the improvement of living standards, how to avoid or cure diseases through diet has become a fashionable way for people because of the side effects brought by drugs. "Medicine food homology" has become a view of returning to green and healthy life in today’s society, which indicated that foods can also be used as drugs. By 2020, there are 101 kinds of homologous substances in China, which are rich in various active ingredients and have the functions of anti-oxidation [1,2], hypoglycemic [3], hypolipidemic [4], and anti-tumor factors. Flavonoids as abundant chemical components in food and medicine homologous substances have attracted widespread attention, owing to the important impact on its quality, function, and safety. They are secondary metabolites of higher plants and most of them are combined with sugars to form glycosides or carbon sugar groups in plants. Hypericin, quercetin, kaempferol, puerarin, luteolin, and rutin are common flavonoids compounds, which are widely found in mulberry leaf, hawthorn, ginkgo, honeysuckle, perilla, and other food and drug homologous substances. A large number of studies have shown that these flavonoid compounds can protect the liver, reduce blood fat and cholesterol, improve the immune function of the body, and promote the body’s health. Besides nutritional value, flavonoids also have functions in prevention and treatment of disease and many other healthcare effects [5]. For example, quercetin has been proved to possess anti-cancer activity in vitro and in vivo. Soo et al. [6] founded that the anti-cancer efficacy of quercetin was determined by inhibiting vascular endothelial growth factor mediated angiogenesis in tamoxifen -resistant breast cancer cells. In the Zhang’s study [7], they concluded that the co-treatment of quercetin and paclitaxel could significantly inhibit the proliferation of prostate cancer cells and increase their apoptosis, playing a beneficial therapeutic effect. 

Considering the great significance of identifying and quantifying of flavonoids in food and drug homologous substances to evaluate their quality and physiological functions, many analytical methods including high performance liquid chromatography (HPLC), ultraviolet spectroscopy (UV), and near-infrared spectroscopy (NIR) have been used [8]. These methods are characterized by expensive detection equipment, complicated and time-consuming procedures, and professional testing personnel [9,10]. In addition, the concentration of flavonoids in edible and medicinal samples must be controlled, which is urgent to develop more sensitive and selective methods for their detection. In recent years, electrochemical sensing technology has been widely used in biological analysis, clinical diagnosis, and other fields due to its high selectivity, high detection efficiency, simple operation, and low cost. The chemical structure of flavonoids determines its properties, and the antioxidant and free radical activities of flavonoids are closely related to its redox behavior. Since the mechanism of its redox is proton-electron transfer, the detection of flavonoid compounds by an electrochemical method can effectively explain the mechanism including stability, antioxidant activity, and promotion of antioxidant action. Compared with the traditional methods, the electrochemical determination of flavonoids has the advantages of wide dynamic linear range, high efficiency, and fast response to electron transfer and low power consumption [11]. For example, Yu et al. [12] reported a facile strategy for quercetin analysis by directly using a bare glassy carbon electrode as the signal sensing platform. The method showed satisfactory results together with a high linear coefficient and a low detection limit of 3.1 nM.

In order to improve the sensitivity of electrochemical flavonoid sensors, modified materials including carbon nanomaterials, metal nanoparticles, ionic liquids, and their combinations were commonly used, which are precisely designed for selective interaction with target analytes and signal amplification [13]. Since the sensing elements could provide more active sites, increase the electrochemical active surface area, improve the mass transport rate, and accelerate the electron transfer rate, good and reliable results were obtained. For example, a new electrochemical platform based on a CoFe_2_O_4_ nanoparticles ionic liquid nanocomposite was reported by Mehmet et al. for rutin analysis, which displayed a satisfactory result owing to high conductivity and fast mobility of ionic liquids [14]. Specifically, carbon-based nanomaterials such as carbon nanotubes, graphene, carbon, and graphene quantum dots, mesoporous carbon, and carbon black have attracted extensive attention in the fabrication of electrochemical flavonoid biosensors because of their excellent electrical conductivity, good biocompatibility, and high durability in this field. This paper is focused on the development of electrochemical sensors based on carbon-based nanomaterials for determining flavonoids and the prospect of their quality and safety evaluation for food and drug homologous substances. 

## 2. Working Principle of the Electrochemical Sensor 

**Figure 1:**
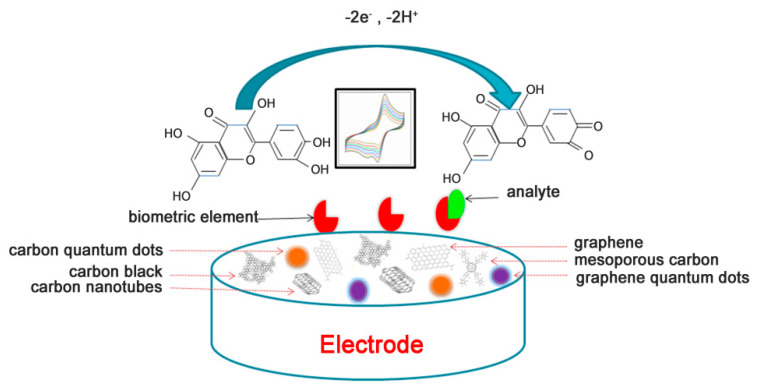
A schematic of the electrochemical biosensor for detecting flavonoids.

The electrochemical biosensor is composed of three parts: biometric identification element, signal converter, and data analyzer. The electrode is the conversion element and the fixed carrier. In addition, the bio-recognition element such as antibodies, enzymes, and cells are fixed to the surface of the electrode by a chemical or physical method. Due to the specific recognition between the bio-identification element and the molecule to be tested, the signal converter converts the target molecule and its response signal into electrical signals, such as capacitance, current, potential, conductivity, and so on. Thus, the qualitative or quantitative detection of the target analyte can be realized. To date, electrochemical detection techniques include differential pulse voltammetry (DPV), cyclic voltammetry (CV), linear sweep voltammetry (LSV), electrochemical impedance spectroscopy (EIS), and square wave voltammetry (SWV) have been employed widely. The working principle of electrochemical sensors based on carbon nanomaterials of flavonoids in food and drug homologous substances was shown in Figure 1.

## 3. Application of Carbon Nanomaterials in the Determination of the Flavonoids in Food and Drug Homologous Substances 

**Figure 2:**
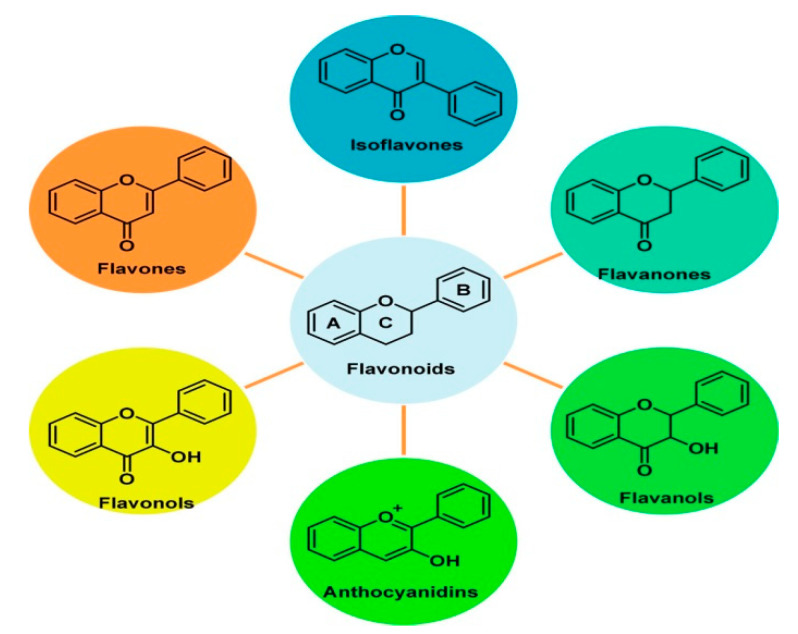
Structures of flavonoids and their subgroup classification. Reproduced with permission from [16], American Chemical Society, 2019.

Carbon-based nanomaterials for electrochemical sensor construction have great properties such as high chemical stability, high thermal and electrical conductivity, and high surface-volume ratio [15]. Flavonoid compounds are derived from the 2-phenyl chromone as the parent nucleus, that is, a series of compounds with C6-C3-C6 as the basic carbon shelf. The mother nucleus of natural flavonoid compounds usually contains hydroxyl, methoxy, hydrocarbon oxygen, isoprene oxygen, and other substituents. As shown in Figure 2, flavonoids are divided into six subunits of flavones, isoflavones, flavanols, flavanols, flavanols, and anthocyanins due to the location and the difference of substituents [16]. The structure of the flavonoid is closely related to its redox properties. In recent years, a number of studies have shown that nanostructured electrochemical platforms comprising of carbon nanotubes, graphene, carbon and graphene quantum dots, mesoporous carbon, and carbon black have been proposed to detect flavonoids in food and drug homologous substances. In this review, electrochemical sensors constructed by carbon-based nanomaterials are reviewed in detail for detecting flavonoids in nearly four years. The materials used and electrochemical properties of the sensors introduced in this paper were summarized as shown in Table 1.

### 3.1. Carbon Nanotubes

Carbon nanotubes (CNTs) are composed from sp^2^ carbon units and present a special structure with nanoscale radial dimensions, micron scale axial dimensions, and a basically sealed tube at both ends [17]. They are mainly composed of carbon atoms arranged in a hexagonal shape to form a coaxial round tube with several to dozens of layers, which can be divided into multi-walled carbon nanotubes (MWCNTs) and single-walled carbon nanotubes (SWCNTs) [2]. CNTs have been widely used in the field of electrochemical sensing because they are easy to achieve surface functionalization and composite with other nanomaterials, thereby improving catalytic efficiency, enhancing surface activity, and promoting the addition of functional groups.

A palladium phthalocyanine-MWCNTs-Nafion modified electrode was developed by Xing et al. [18] to enhance sensitive electrochemical detection of rutin. The biosensor exhibited reproducible results with a wide linear range from 0.1 to 51 μM and a low-limit detection of 75 nM, owing to the excellent electrocatalytic activity of MWCNTs and palladium phthalocyanine. In addition, the sensor is expected to be widely used in traditional Chinese medicine analysis or quality monitoring of actual samples since the common co-existing ions have no interference with the determination of rutin. Meanwhile, Jorge A. and coworkers [19] constructed a nanostructured biosensor for rutin based on a new combination of SWCNT, chitosan, and neodymium(III) oxide (Nd_2_O_3_). The modified electrode surface was characterized by CV and EIS, while the quantification of rutin in real samples was measured by SWV. The proposed sensor could effectively detect rutin at concentrations below 0.1 μM.

It is well known that functional modification of carbon nanotubes can effectively improve their dispersibility in solvents and broaden their application fields. For example, Yang et al. [20] used helical carbon nanotubes (HCNTs) and gold nanoparticles (AuNPs) to fabricate a sensor due to its unique 3D helical structure and excellent conductivity of HCNTs, the superior stability, and sensitivity of AuNPs. In this work, HCNTs were functionalized with Poly(diallyl dimethyl ammonium chloride) and AuNPs were immobilized on PDDA-HCNTs by electrostatic interactions. The method showed satisfactory results with a wide linear response range and a low detectable limit of 81 nmol/L for determining rutin. The facile electrochemical biosensor was developed by Liang et al. [21] based on multi-walled carbon paste electrode (MWCPE) to load the composite materials of cetyltrimethyl ammonium bromide and carboxylic multi-walled carbon nanotubes (CTAB-cMWCNTs). Owing to effective electrochemical performance together with outstanding stability and sensitivity, the CTAB-cMWCNTs/MWCPE was triumphantly applied to determine kaempferol and quercetin, respectively, in real samples and obtained favorable results. Likewise, Liu et al. [22] employed a DPV method to study the simultaneous determination of myricetin and rutin on cMWCNT-pTh-Pt modified glassy carbon electrode(CGE), which obtained extremely satisfactory results with a 0.01–15 μM wide linear response range and the detection limits were 3 nM and 1.7 nM, respectively. In another study, the application of CNTs-based biosensor was proposed for the detection of rutin in orange, oat, salvia, and other real samples [23]. The results demonstrated that the sensor has high reproducibility and sensitivity. A novel method based on the DPV assisted by chemometrics was employed for quercetin detection in the presence of tannic acid [24]. The research showed that two overlapping voltammetric peaks were founded when using a carbon paste electrode modified with MWCNTs to detect samples, but this problem was effectively solved by chemometrics. The proposed sensor was successfully applied in real samples. In another report, Rajabi et al. [25] developed a novel electrochemical sensor for detecting quercetin using a large pass ion-exchange nanoresin-MWCNT nanocomposites with the ranges of 1.8–570 μM, meanwhile the detection limit reached as low as 0.213 μM. Moreover, the GCE surface modification with MWCNT and poly-gallic acid were used to construct a sensor for measuring the quercetin voltammetric response [26]. As the results demonstrated, the proposed sensor could effectively detect the quercetin of medicinal herb extracts and showed high selectivity in the presence of other phenolics.

**Figure 3:**
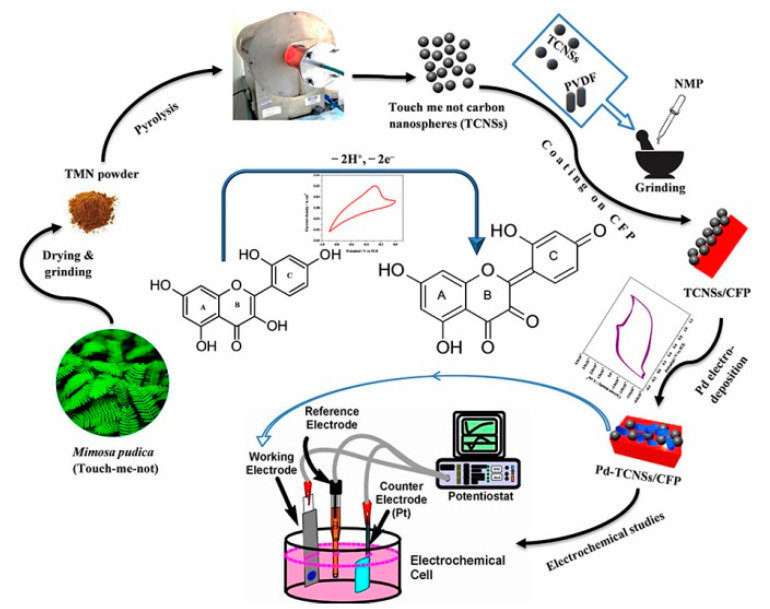
Synthesis schematic diagram of Pd-TCNSs/CFP. Reproduced with permission from [28], American Chemical Society, 2020.

With the continuous development of electrochemical technology, researchers have begun to study other flavonoids. An electrochemical sensor for detecting flavonoid morin was proposed based on NH_2_-MWCNT/ZnO composites [27]. Using CV and DPV as the transduction techniques, the authors discussed different analysis parameters such as accumulation time, potential, and others to influence the property of the sensor. The results revealed a low limit of detection (LOD) of 0.002 μM, which was attributed mainly to the predominant catalytic activity of the modified screen printed carbon electrode (SPCE). Especially, as shown in Figure 3, Chaithra et al. [28] proposed a method to synthesize the nanocomposite using palladium nanoparticles electrodeposited on carbon nanospheres for the sensitive detection of morin. The carbon nanospheres along with Pd improves the electrode–electrolyte interfacial property so that electrical conductivity is enhanced and electronic transfer is accelerated. The fabricated sensor showed favourable results and could determine morin in actual samples such as mulberry. In addition, Tugçe et al. [29] constructed a new sensitive sensing platform based on CNTs-niobium nanoparticles for chlorogenic acid analysis. In this paper, the voltammetric behaviour of chlorogenic acid was analyzed by CV and the proposed platform provided favourable results in various food samples.

### 3.2. Graphene

Graphene’s characteristics of high surface area, high conductivity, great electrochemical, thermal and mechanical stability, tunable electrical properties, and large potential functional areas made it widely used in electrochemical sensing applications. The hydrophobic nature of graphene (GO) makes it liable to form nonreversible aggregation and even restack due to the van der Waals force and the strong π-π stacking interaction [9,30]. Therefore, functional reagents are often needed to functionalize graphene to improve its suitability. There are now substantial research studies that have shown that graphene can be utilized as a part of composite electrode structures to improve the sensing performance [31,32].

The papers of electrochemical sensors for detecting puerarin are abundant. Jing et al. [33] proposed a method to synthesize the nanocomposite of graphene, poly(sodium 4-styrenesulfonate) (PSS), and WO_3_ nanorods for the sensitive detection of puerarin with satisfactory consequences. A similar assay using the graphene-based sensors directed toward puerarin was shown superior stability and recovery in real samples [34]. Recently, Sheng et al. [35] constructed a voltammetric sensing platform using polyethyleneimine-functionalised graphene (PEI-GR). In their study, the proposed sensor showed excellent electrochemical behaviour with a lower detection limit of 8 × 10^−8^ mol L^−1^ and a large linear detection range (3 × 10^−7^–1 × 10^−5^ mol L^−1^), which was appropriate to detect puerarin in practical samples. Molecular imprinting is an effective method to detect puerarin. For example, Li and coworkers proposed a novel electrochemical platform using molecularly imprinted polymer (MIP) and reduced graphene oxide (rGO) [36]. In this case, puerarin was used as a template molecule, while o-phenylenediamine (o-PD) acted as a functional monomer. After the removal of puerarin template, specific binding sites of puerarin were formed in the MIP matrix so that a significant redox peak could appear in subsequent detection. This method had the advantages of rapid response time, high sensitivity, outstanding selectivity, and low cost, providing a new possibility for puerarin detection in a wide variety of samples. Equally, a daidzein templated MIP electrochemical sensor was developed via the electropolymerization of o-PD on the surface of PSS-rGO/GCE [37]. The results demonstrated that the sensor has good anti-interference ability and sensitivity, which can be used in natural products and medicinal samples.

It is necessary to detect rutin and quercetin sensitively because they exist in many food and drug homologous substances, and it is an important component of many clinical drugs [38]. In Li’s work [39], a sensitive sensor was successfully fabricated by using the compound of Cu_2_O, Au nanoparticles, and nitrogen-doped graphene (Cu_2_O-Au/NG), which achieved the linear calibration range for rutin was 0.06–512.9 μM with a lower detection limit of 30 nM. Afterward, some studies have suggested that increasing the content of N in graphene can provide richer binding sites for non-covalent functionalization, enhance the density of free carriers and conductivity, and, thus, improve the biocompatibility and sensitivity in the application of biosensing. Yang et al. [40] have fabricated novel Au-Ag nanothorns assembled on N-doped graphene (NG), which showed a brilliant electrochemical property toward oxidation of rutin. A analogical research using SnO_2_ nanoparticles assembled on NG for rutin determination was shown desirable results with a low detection limit of 0.2 nM [41]. Particularly, Niu et al. [42] proposed a 3D-reduced graphene oxide aerogel (RGA) material with porous skeleton for the detection of quercetin. Compared to the traditional RGA, the newly synthesized 3D-RGA showed considerable improvement of properties due to its unique porous skeleton, greater surface area, and outstanding conductivity. The new biosensor managed to detect quercetin as low as 0.065 μM in Ginkgo tablet samples.

**Figure 4:**
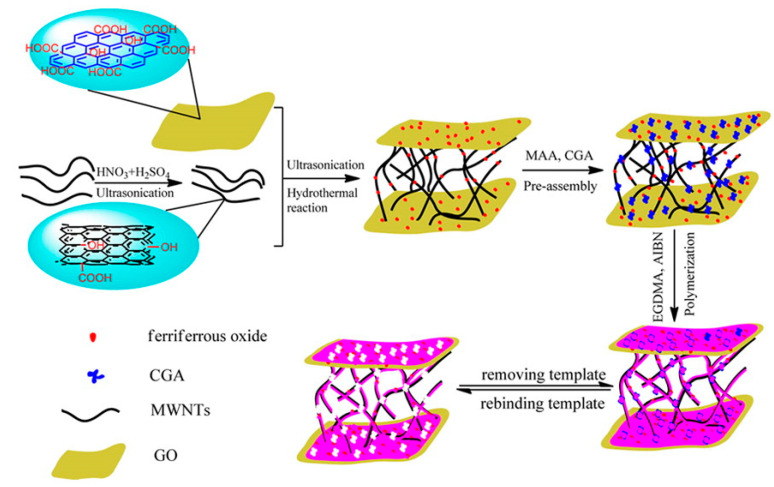
Synthesis schematic diagram of 3D MMIPs. Reproduced with permission from [43], American Chemical Society, 2016.

In recent years, graphene and carbon tube composites have been widely used in detecting flavonoids. For instance, Wang et al. [43] fabricated a 3D graphene-MWCNTs network and applied it to the detection of hyperin, which demonstrated a good linear from 0.005 to 1.5 μM with a LOD of 0.001 μM. As shown in Figure 4, a novel 3D magnetic imprinted polymer (MMIP) with chlorogenic acid as a template molecule and methacrylic acid as a functional monomer was prepared using a new graphene-carbon nanotube composite as a carrier. In this study, the 3D MMIPs have been applied in real samples successfully and possessed good selectivity and sensitivity toward chlorogenic acid [44]. Furthermore, a ternary Pt-rGO-MWCNTs nanocomposite was exhibited by Satar et al. to establish the electrochemical sensor of the simultaneous measurement of myricetin and rutin [45]. Electrical signals with myricetin and rutin were characterized by DPV, displaying reliable results with excellent reproducibility and stability in practical samples.

### 3.3. Carbon and Graphene Quantum Dots

Quantum dots including carbon quantum dots (CQDs) and graphene quantum dots (GQDs) are considered one of the most promising carbon-based nanomaterials in the field of electrochemical sensing [46]. They not only could be modified by absorbent surface chemicals, but also have many characteristics including lower toxicity, outstanding electronic properties, large specific surface area, and a large number of functional site [47,48]. To date, researchers have devised plenty of researchers have designed a number of methods to detect flavonoids using carbon and graphene quantum dots. 

Meng et al. [49] constructed an electrochemical sensor based on GQDs and poly (3, 4-ethylenedioxythiophene) (PEDOT) bilayer films by two-step electrodeposition and the electrochemical property was characterized by CV. The results proved that the GQDs/PEDOT/GCE exhibited a strong electrical response toward rutin redox due to the high surface area of the GQDs. Another label-free and selective electrochemical biosensor was developed by Tang et al. [50] based on GQDs and Au nanoparticle nanocomposites. Herein, the oxidation current of luteolin measured with the newly synthesized electrode was 16 times higher than that of bare GCE. In addition, the relationship of the oxidation current vs. concentration was shown to be linear in the range of 0.001–10 μM. This method provided more possibilities to detect luteolin in pragmatic applications. Zhao et al. [51] have firstly presented an application of MoS_2_-CNTs@graphene oxide nanoribbons, thiol-beta-cyclodextrin, and GQDs nanocomposite modified GCE for detecting quercetin by CV. In this study, this modified electrode displayed an ultra-low LOD of 8.2 × 10^−4^ μM so that the sensor might have great potential in the detection of more actual samples. Recently, Zhou et al. [52] introduced an ultra-sensitive electrochemical determination of quercetin by using the nanocomposite of thiolated β-cyclodextrin functionalized Au nanoparticles (Au-β-CDs) and protonated aminated GQDs (NH_2_-GQDs). The quercetin sensor possessed a good molecular recognition effect on target molecules, and favorable selectivity in the determination of honeysuckle in human serum samples. For determining rutin, Cu-doped carbon quantum dots (Cu-CQDs) were developed [53]. The linearity range for the detection of rutin was from 0.1–15 μg mL^−1^ with a low detection limit of 0.05 μg mL^−1^.

### 3.4. Mesoporous Carbon

With the development of carbon-based nanomaterials, mesoporous carbon (MC) have aroused remarkable interest in electrochemical detection fields because of distinct structural characteristics such as a large active surface area and pore volume, great conductivity, and outstanding stability in the terms of mechanical and thermal factors [54,55]. Some papers have been proved that mesoporous carbon can be used as a favorable position for electron transfer and adsorption position in electrochemical sensors owing to its vacancy defects [56,57]. Therefore, more researchers have prepared electrochemical sensors using mesoporous carbon as a recognition element [58].

In order to detect rutin, Nourali et al. [59] have fabricated a novel modified carbon paste electrode by using defective mesoporous carbon (DMC) and room temperature ionic liquid 1-butyl-3-methylimidazolium hexafluorophosphate (RTIL BMIM•PF6) and then investigated their electrochemical behaviors via chronocoulometry, CV, and EIS. The resulting biosensor was shown excellent applicability in various real samples. Xu’s team prepared a Pd/MoS_2_ and ionic liquid functionalized ordered mesoporous carbon (IL-OMC) composite using a chemical reduction strategy and evoluted the electrochemical performance of quercetin by using this material [60]. Hence, the sensor exhibited remarkable performance with a broad linearity range of 0.02–10.0 μΜ and a low detection limit (8.0 nM). At present, Metal organic framework (MOF) have great potential in the field of electrocatalysis. For example, the MC and zirconium fumarate metal-organic framework (MOF-801) composite was applied to detect gallic acid and luteolin for the first time [61]. In general, the proposed material significantly improved the conductivity and electrocatalytic performance of the sensor. Another electrochemical biosensor with the MOF structure was established by Zhao et al. and they discussed the electrochemical behaviors of chlorogenic acid [62]. The biosensor showed a rapid electrochemical response, continuous stability, and good sensitivity in a real pharmaceutical sample. Subsequently, Ahmet et al. [63] reported a nanocomposite of the Co nanoparticle decorated by micro-mesoporous carbon (Co/ZIF-C) in order to identify rutin with specificity. When the target molecule was captured, the Co ions were released from the Co/IF-C and then the sensing response current could be detected by DPV. The newly prepared sensor demonstrated greater selective and was highly sensitive toward rutin.

### 3.5. Carbon Black 

Carbon black (CB) is an old and low-cost carbon-based material, which exhibits many advantages such as superior electrical conductivity and fast electron transfer dynamics. Moreover, it is easy to be functionalized due to it having a large number of defect sites [64]. Therefore, it has a wide range of applications in developing inexpensive electrochemical devices. 

As shown in Subbiramaniyan’s research [65], a CB/WO_3_ nanohybrid was synthesized by a single-step hydrothermal method, which improved the electrochemical performance of the sensor and could be successfully used for the detection of rutin in real sample analysis. The fabricated sensor showed satisfactory results with the lowest LOD (2 nM) and reliable linearity (0.01–75.46 μM). Then, Tugçe et al. [66] developed a new electrochemical strategy to detect chlorogenic acid using an electrode modified with CB. Using SWV as the transduction technique, the authors studied the electrochemical properties. The results revealed a low LOD of 4.1 × 10^−9^ M, which was attributed mainly to the predominant catalytic activity of the modified boron doped diamond electrode.

## 4. Conclusions

To sum up, we summarized the latest research progress of electrochemical sensors based on carbon nanomaterials including carbon nanotubes, graphene, carbon and graphene quantum dots, mesoporous carbon, and carbon black in detecting the bioactive components of flavonoids in food and drug homologous substances over the past four years. As bioactive ingredients, flavonoids can not only be used to make drugs to prevent and treat diseases such as diabetes and cell tumors, but can also be used as dietary supplements to keep healthy. On the other hand, the content of flavonoids has a great impact on its safety. From the perspective of dietary supplement and pharmacological application, it is of great importance to establish a simple and sensitive method for determining flavonoids content. 

To date, electrochemical sensors based on carbon nanomaterials have been widely reported for the analysis of real samples, especially for the detection of quercetin and rutin. Generally, carbon nanomaterials with high specific surface area, excellent electrical conductivity, good stability, and unique mechanical properties so that they can accelerate the electron transfer reaction and the catalytic reaction of the electrode surface. In addition, carbon-based nanomaterials can improve their electrochemical properties by functionalizing or combining with metal ions and organic metal skeletons. Most of the constructed sensors significantly improved the detection range of flavonoid compounds and reduced the detection limit, laying a foundation for further research on detecting active ingredients. Despite these advances, there are still some challenges in detecting flavonoids in food and drug homologous substances, such as incomplete separation of components, interference of similar components, and instability of electrochemical sensors. The development of electrochemical sensors has some limitations when compared with the existing high-performance liquid chromatography-photodiode array detectors that can simultaneously quantify multiple flavonoid compounds. To solve the critical issues, further research is needed. We herein speculate a few prospects. First, some strategies could be used to design and develop novel nanomaterials with an ideal size and shape and contain the best active sites, thus improving the selectivity of the sensors. With the development of molecular biology technology, more antibodies or aptamers can be synthesized to capture target analytes with no or weak electrochemical activity in food and drug homologous substances in order to improve the specificity and application range of electrochemical sensors. Then, it is necessary to isolate and purify bioactive components from the background matrix of food and drug homologous substances or greatly improve the electrocatalytic performance of the sensor in order to realize the sensitive detection of flavonoids in the actual samples. Finally, it is necessary to develop multiple array electrodes to detect a variety of compounds simultaneously and it is urgent to develop micro or portable electrochemical devices to improve their applicability.

**Table 1:** Comparison of electrochemical sensors based on carbon nanomaterials for determining flavonoids in medicine food homology.

Carbon Nanomaterial	Analyst	Detection Technique	Linear Range (µM)	Detection Limit (µM)	References
-	quercetin	DPV	0.1–15	0.003	[12]
CNTs	rutin	CV	0.10–51	0.075	[18]
CNTs	rutin	SWV	0.99–8.0	0.092	[19]
CNTs	rutin	CV	0.10–31	0.081	[20]
CNTs	kaempferol, quercetin	DPV	5.00–50 5.00–20	0.012 0.005	[21]
CNTs	myricetin, rutin	DPV	0.01–15 0.01–15	0.003 1.7 × 10^−3^	[22]
CNTs	rutin	DPV	0.01–10	1.8 × 10^−3^	[23]
CNTs	quercetin	DPV	0.005–0.6	1.96 × 10^−3^	[24]
CNTs	quercetin	CV	1.8–570	0.213	[25]
CNTs	quercetin	CV	0.075–100	0.054	[26]
CNTs	morin	DPV	0.2–803.4	0.002	[27]
CNTs	chlorogenic acid	SWV	0.002–2.0	8.2 × 10^−4^	[29]
graphene	puerarin	CV, LSV	0.06–6.0	0.04	[33]
graphene	puerarin	CV	0.23–5.5	0.04	[34]
graphene	puerarin	LSV	0.3–10	0.08	[35]
graphene	puerarin	CV	0.02–40	0.006	[36]
graphene	daidzein	CV	0.001–0.02	5.0 × 10^−4^	[37]
graphene	rutin	CV, DPV	0.06–512.9	0.03	[39]
graphene	rutin	DPV	0.1–420	0.015	[40]
graphene	rutin	DPV	0.4–2	2.0 × 10^−4^	[41]
graphene	quercetin	DPV	0.1–100	0.065	[42]
CNTs-graphene	hyperin	CV	0.005–1.5	0.001	[43]
CNTs-graphene	myricetin, rutin	DPV	0.05–50 0.05–50	0.01 0.005	[45]
GQDs	rutin	CV, EIS	0.05–10	0.011	[49]
GQDs	luteolin	DPV	0.01–10	0.001	[50]
GQDs	quercetin	DPV	0.002–1.6	8.2 × 10^−4^	[51]
GQDs	quercetin	CV	0.001–0.2	2.85 × 10^−4^	[52]
CQDs	rutin	Fluorescent	0.1–15	0.05	[53]
mesoporous carbon	rutin	SWV	0.008–4	1.17 × 10^−3^	[59]
mesoporous carbon	quercetin	LSV	0.02–10	0.008	[60]
mesoporous carbon	luteolin	CV	0.02–10	2.9 × 10^−3^	[61]
mesoporous carbon	chlorogenic acid	DPV	0.1–15	0.019	[62]
mesoporous carbon	rutin	CV, DPV	0.1–30	0.022	[63]
CB	rutin	CV, DPV	0.01–75.46	0.002	[65]
CB	chlorogenic acid	SWV	0.02–2	4.1 × 10^−3^	[66]
